# Predictive value of plasma sB7-H3 and YKL-40 in pediatric refractory Mycoplasma pneumoniae pneumonia

**DOI:** 10.1515/med-2024-1114

**Published:** 2025-01-15

**Authors:** QiuMin Zhao, ShiYan Ji, HaiPing Jiang, DongMing Lu, LiFen Qian, JingWen Zhang, Yue Cui, Wei Lin, HaoYing Ge, Meng Gu

**Affiliations:** Department of Laboratory Medicine, Changzhou Children’s Hospital of Nantong University, Changzhou, Jiangsu, 213003, China; Children’s Health Research Center, Changzhou Children’s Hospital of Nantong University, Changzhou, Jiangsu, 213003, China; Department of Respiratory, Changzhou Children’s Hospital of Nantong University, Changzhou, Jiangsu, 213003, China; Department of Laboratory Medicine, Changzhou Children’s Hospital of Nantong University, No. 958, Zhongwu Avenue, Diaozhuang Street, Tianning District, Changzhou, Jiangsu, 213003, China

**Keywords:** Mycoplasma pneumoniae pneumonia, refractory mycoplasma pneumoniae pneumonia, children, sB7-H3, YKL-40

## Abstract

**Objective:**

This study investigated the clinical significance of plasma sB7-H3 and YKL-40 levels in children with refractory Mycoplasma pneumoniae pneumonia (RMPP).

**Methods:**

A total of 182 RMPP patients (103 general Mycoplasma pneumoniae patients and 79 RMPP patients) were included. sB7-H3, YKL-40, and other inflammatory factors were measured. Independent factors associated with the early diagnosis of RMPP were determined. The value of each independent risk factor in predicting RMPP was evaluated.

**Results:**

The RMPP group reported significantly longer hospital stays and total fever durations. Levels of C-reactive protein, D-dimer, IL-13, IL-6/-10, sB7-H3, and YKL-40 were higher in the RMPP group. sB7-H3 was positively correlated with IL-13, IL-6, and IL-4, whereas YKL-40 was positively correlated with all of the above indicators (IL-5 was also included). sB7-H3 and YKL-40 were independent risk factors for RMPP. The critical values for sB7-H3 and YKL-40 were 3.525 and 313.3 ng/mL, respectively. sB7-H3 and YKL-40 had areas under the curve of 0.734 and 0.859, respectively. YKL-40 had high sensitivity and specificity of 88.61 and 87.38%, respectively. Both indicators had predictive value, YKL-40 had the highest predictive ability for RMPP.

**Conclusion:**

Detection of sB7-H3 and YKL-40 levels in the plasma may be useful in diagnosing RMPP early in the disease process.

## 1 Introduction

Mycoplasma pneumoniae pneumonia (MPP) is one of the complex manifestations of Mycoplasma pneumoniae (MP) infection and is a pathogen responsible for community-acquired pneumonia (CAP) in children [1]. Some children require hospitalization for MP infections, but the majority are mild and self-limiting [2]. MPP accounts for 10–40% of pneumonia in hospitalized children [3]. Although most patients with MPP respond to macrolide antibiotic therapy, some patients with MPP may develop a complicated disease process that may even be life threatening [4,5]. Refractory Mycoplasma pneumoniae pneumonia (RMPP) results in persistent fever and extrapulmonary system involvement due to abnormal immune response, MP resistance, and mixed infections [6,7].

Macrolides are the antibiotics of choice for pediatric patients [8]. In the last decade, RMPP has been on the rise and remains unresolved despite macrolide therapy [9]. It is generally accepted that patients who continue to have the same clinical signs/symptoms, persistent fever, and unchanged lung imaging after 7 or more days of macrolide antibiotic therapy may be considered to have RMPP [10]. Even persistent exacerbation of RMPP can be complicated by pleural effusion, pulmonary atelectasis, pneumomediastinum, and necrotizing pneumonia [11,12]. Currently, RMPP is mainly caused by direct invasion of MP into lung and bronchial tissues or by MP stimulation of excessive immune-inflammatory responses *in vivo*, including strong expression of cytokines and highly activated cell-mediated immune responses [9,13]. In addition, the pathogenesis of MP infection is closely related to the activation of macrophages through the release of immunomodulatory and inflammatory cytokines [14,15]. Following pathogenic stimuli or cytokine activation, these cells typically migrate to areas of inflammation, differentiate into macrophages, and orchestrate local responses by expressing cytokines that attract and activate other cells [16,17].

B7-H3 is a member of the B7 superfamily and is widely expressed in human tissues [18]. B7-H3 is closely associated with the onset and severity of respiratory diseases such as lung cancer, bronchial asthma, and pneumonia. With further research, plasma soluble B7-H3 (sB7-H3) levels in the above diseases are higher than those in the normal population. sB7-H3 is expressed in activated T cells, monocytes, and dendritic cells [19]. sB7-H3 is a major inflammatory mediator in the pathogenesis of MP infections, and promotes the secretion of inflammatory factors by Th2 cells through its involvement in the TLR4/NF-κB signaling pathway IL-5, IL-4, which aggravate inflammatory injury in lung tissues [20,21]. sB7-H3 has been demonstrated to be significantly increased in pediatric patients with MP [22].

YKL-40 is a chitinase analog protein found in mammals and is a normal component of human plasma [23]. YKL-40 has been reported to promote the maturation of monocytes to macrophages and is subsequently secreted mainly by mature macrophages [24]. Many studies have shown that YKL-40 is highly expressed in a variety of inflammatory diseases and that YKL-40 plays an important role in determining disease severity and prognosis of regression [25–27].

Currently, the current pathogenesis of CAP in children relies on the level of biomarker testing [28–31]. Despite the extensive clinical experience with these markers, they are still insufficient to differentiate RMPP from MPP, and while new biomarkers are constantly being discovered, early identification of RMPP remains challenging. Therefore, there is a need to identify specific and sensitive biochemical indicators for the early diagnosis of RMPP.

By studying and comparing differences between MPP and RMPP cases, this study was aimed at identifying independent factors associated with RMPP, and to provide a basis for early clinical diagnosis of RMPP.

## 2 Materials and methods

### 2.1 Patients

This prospective single-center study was conducted from March 2022 to December 2023 at Changzhou Children’s Hospital of Nantong University. All patients presented with signs and symptoms of pneumonia on admission, including fever, cough, abnormal lung auscultation, and infiltrates on chest radiographs. The diagnosis of MP infection in all patients was confirmed by plasmapheresis tests (MP anti-MP-IgM positive and antibody titer ≥1:160) and/or polymerase chain reaction detection of positive MP results in throat swab specimens [32]. A total of 182 children with a final diagnosis of MPP were hospitalized.

**Figure 1:**
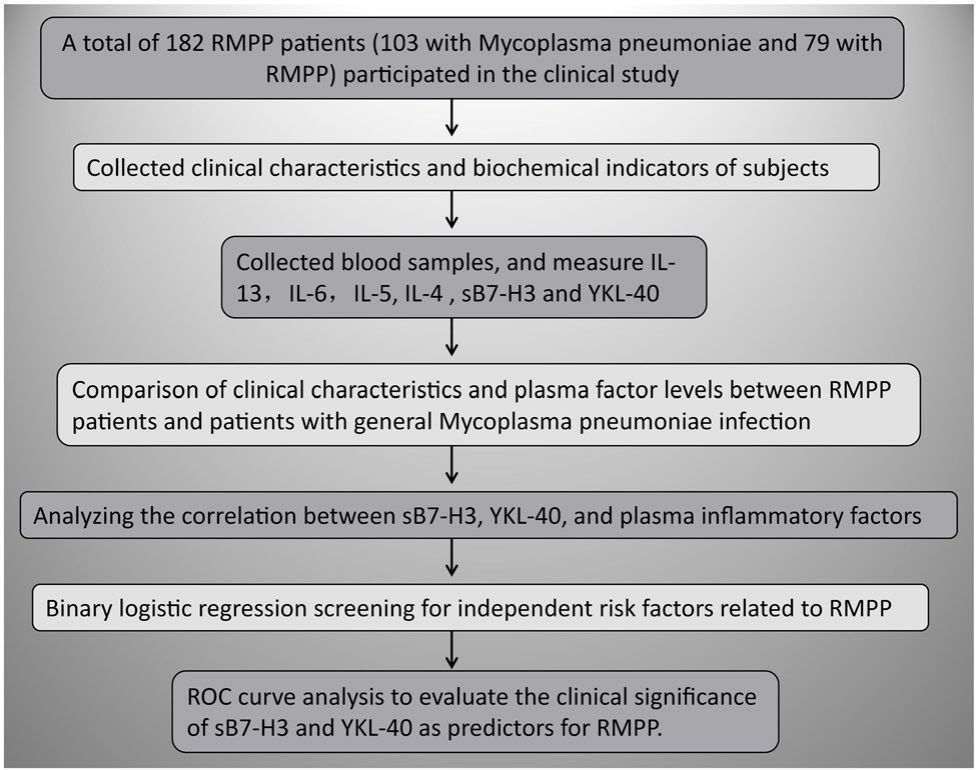
Study flow diagram.

Based on the diagnostic criteria (exacerbated clinical symptoms, persistent fever [≥38.5℃], and worsening pulmonary imaging despite 7 days of treatment with macrolide antibiotics), patients were categorized into either the RMPP group or general Mycoplasma pneumoniae (GMPP) group. Cases diagnosed with RMPP were investigated appropriately to exclude other pathogens such as pneumococcus or mixed infections. Children were excluded if they had proven congenital heart disease, chromosomal disorders, metabolic disorders, immunodeficiency diseases, chronic lung disease, or asthma. Finally, 103 patients with GMPP and 79 with RMPP were included. Study flow as shown in Figure 1.

### 2.2 Clinical features and biochemical indicators

Demographic characteristics, including age, gender, total fever duration, and medical history, were collected from the subjects’ records. Upon admission, laboratory tests were performed within 24 h and the results were recorded. Fever duration: the time of fever onset was obtained from the patient’s guardian, and measurements were taken by the healthcare provider every 4–6 h after admission to record the time from the fever onset until the temperature returned to normal completely. Fever was defined as a patient’s temperature ≥38.5°C measured with an electronic thermometer or mercury thermometer under standard operating conditions.

### 2.3 Prognosis

The endpoint was set as progression to severe MPP during in-hospital treatment, and children diagnosed with severe MPP on admission were excluded. The incidence of eventual progression to severe MPP in the GMPP and RMPP groups was observed as well as plasma factor levels in children with mild and severe MPP. (1) Mild MPP: respiratory rate <70 breaths/min at age <3 years or respiratory rate <50 breaths/min at age ≥3 years, normal feeding, and no dehydration. (2) Severe MPP: respiratory rate ≥70 breaths/min at age <3 years, or respiratory rate >50 breaths/min at age ≥3 years, cyanosis, nostrils flared and visibly retracted, anorexia, and dehydration. (3) MPP in combination with pleural effusions, pulmonary necrosis/pulmonary abscess. (4) MPP combined with other systemic dysfunction.

### 2.4 Measurement of routine blood and biochemical indicators

Routine blood tests were performed using a Sysmex XN-1000 hematology analyzer (Sysmex Co., Kobe, Japan) to detect white blood cell (WBC) count, neutrophil (NEP), neutrophil/lymphocyte ratio (NLR), platelet (PLT) count, and albumin. A fully automated chemistry analyzer (Japan) was used to determine C-reactive protein (CRP) level, alanine aminotransferase (ALT) level, aspartate aminotransferase (AST) level, and lactate dehydrogenase (LDH) level.

### 2.5 Plasma factor measurements

Enzyme-linked immunoassay kits were used on admission to detect sB7-H3 (Rapidbio, Hayward, CA, USA); YSK-40 (Quidel, San Diego, CA, USA); and IL-13, IL-6, IL-5, and IL-4 (Nanjing Jiancheng Biological Company, Nanjing, China). Each patient’s imaging data and laboratory test data were reviewed by appropriate specialized physicians who had no knowledge of the subject’s information.

### 2.6 Data statistics

Statistical analyses were performed using SPSS 20.0 software. The Shapiro–Wilk test was used to determine the normality of the data. Measurement data are shown as mean ± standard deviation, and Student’s *t*-test was used for data on continuous variables in a normal distribution. Mann–Whitney *U*-test was used for data on continuous variables in a skewed distribution. Count data were expressed as frequencies and ratios and Chi-square test was used. Spearman’s test was used to analyze the interrelationships between plasma factors and Bonferroni correction was used for multiple comparisons. Binary logistic regression analyses of risk factors associated with RMPP were performed (variable selection criterion was *P* < 0.05), and the predictive validity of the model for RMPP was subsequently assessed using receiver operating characteristic (ROC) curve and calculation of the area under the ROC curve (AUC). *P* < 0.05 was considered to be statistically significant.


**Informed consent:** Informed consent was obtained from all guardians of the patients.
**Ethical statement:** All procedures performed in this study involving human participants were in accordance with the ethical standards of the institutional and/or national research committee and with the 1964 Helsinki Declaration and its later amendments or comparable ethical standards. The study was approved by the Institutional Human Ethics Committee of Changzhou Children’s Hospital of Nantong University (No. 202003CZ18).

## 3 Results

### 3.1 General and clinical features

There were no significant differences neither in demographics between the RMPP and GMPP groups, including age (*P* = 0.325) and gender (*P* = 0.418) nor in disease characteristics, including lung rales (*P* = 0.329), lung wheezing (*P* = 0.348), and pleural effusion (*P* = 0.325) (Table 1). Patients in the RMPP and GMPP groups were compared with respect to clinical symptoms, biochemical levels, and routine blood markers. There was a difference in the total fever duration (*P* = 0.016). Blood routine showed that the neutrophil percentage (NEP%) was 6.98 ± 3.98 and 5.12 ± 4.69 in patients in the RMPP and GMPP groups, respectively, which was significantly different (*P* = 0.010), whereas there was no significant difference in the NLR and PLT count (*P* = 0.079, *P* = 0.062). In addition, some biochemical indices, such as CRP and D-dimer, were elevated in patients in the RMPP group compared to the GMPP group (*P* < 0.001). The CRP and D-dimer in the RMPP group were 54.32 ± 32.9 mg/mL and 2.12 ± 1.65 μg/mL, respectively. In addition, AST and ALT were not significantly different (*P* = 0.770, *P* = 0.090) between the two groups.

**Table 1:** Clinical characteristics of patients with GMPP and RMPP

Variables	GMPP (*n* = 103) *N* (%)	RMPP (*n* = 79) *N* (%)	*P* value
**General characteristics**			
Age (years)	6.16 ± 2.34	5.79 ± 2.28	0.325
Male/female	47/56 (45.6/54.4)	40/39 (50.6/49.4)	0.503
Hospital stay (days)	7.2 ± 1.9	11.4 ± 2.2	<0.001
**Signs and symptoms**			
Total fever (days)	8.2 ± 1.9	10.3 ± 2.2	0.016
Lung rales, *n* (%)	37 (35.9)	34 (43.0)	0.329
Lung wheezing, *n* (%)	27 (26.2)	16 (20.2)	0.348
Pleural effusion, one/two, *n* (%)	77/26 (74.8/25.2)	51/28 (64.6/35.4)	0.135
**Routine blood**			
WBC (× 10^9^/L)	15.36 ± 14.89	10.36 ± 6.36	0.065
NEP%	5.12 ± 4.69	6.98 ± 3.98	0.010
NLR	2.95 ± 2.03	3.35 ± 2.65	0.079
PLT (× 10^9^/L)	253.3 ± 52.3	286.3 ± 69.36	0.062
Albumin (g/dL)	66.32 ± 4.36	64.36 ± 5.36	0.851
**Humoral immunity**			
Total IgG (g/L)	7.29 ± 3.56	8.06 ± 4.02	0.078
Total IgA (g/L)	0.89 ± 0.69	1.03 ± 0.81	0.25
Total IgM (g/L)	1.24 ± 0.53	1.41 ± 0.59	0.056
**Biochemical indicators**			
CRP (mg/L)	27.02 ± 20.75	54.32 ± 32.9	<0.001
ALT (U/L)	33.89 ± 20.82	40.94 ± 30.12	0.770
AST (U/L)	26.22 ± 21.32	32.09 ± 20.92	0.090
LDH (U/L)	389.75 ± 234.62	403.02 ± 214	0.861
D-dimer (μg/mL)	1.53 ± 1.60	2.12 ± 1.65	<0.001

Note: WBC, white blood cells; NEP, neutrophil ratio; NLR, neutrophil/lymphocyte ratio; PLT, platelets; Ig, immunoglobulin; CRP, C-reactive protein; ALT, alanine aminotransferase; AST, aspartate aminotransferase; LDH, lactate dehydrogenase.

All data are shown as median [IQR] or *N* (%). Categorical values were compared using the chi-square test. Student’s *t*-test or Mann–Whitney test was used to assess differences between the two groups. *P* values of <0.05 were considered statistically significant.

### 3.2 Plasma factor levels and factor correlations

**Figure 2:**
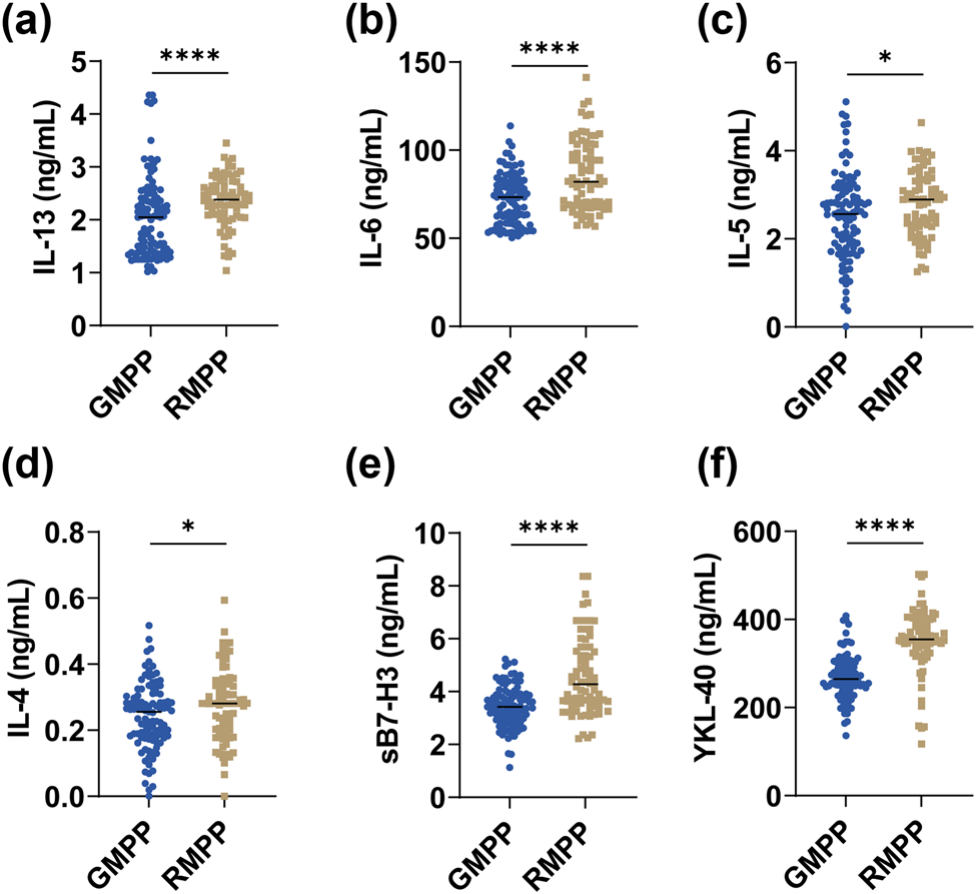
Comparison of plasma factor levels in the GMPP and RMPP groups. (a) IL-13, (b) IL-6, (c) IL-5, (d) IL-4, (e) sB7-H3, and (f) YKL-40 in the GMPP and RMPP groups. *P < 0.05; ****P < 0.0001.

sB7-H3, YKL-40, and inflammatory factors were detected and statistically analyzed. The values of IL-13, IL-6, IL-5, IL-4, sB7-H3, and YKL-40 were significantly higher in the RMPP group than in the GMPP group (*P* < 0.05; Figure 2). Subsequently, the correlation of sB7-H3 and YKL-40 with these inflammatory factors was assessed (Table 2). sB7-H3 showed a moderately strong positive correlation with IL-4 (*R* ^2^ = 0.456, *P* < 0.0001) and a weak positive correlation with IL-13 (*R* ^2^ = 0.249, *P* < 0.0001). YKL-40 showed a moderately strong positive correlation with IL-6 (*R* ^2^ = 0.418, *P* < 0.0001) and IL-4 (*R* ^2^ = 0.329, *P* < 0.0001), and a weak correlation with IL-13 (*R* ^2^ = 0.289, *P* < 0.0001). sB7-H3 and YKL-40 were both not correlated with IL-5 (both *P* > 0.05).

**Table 2:** Correlation analysis between patients’ plasma sB7-H3, YKL-40 and other factors

Variables	sB7-H3		YKL-40	
	*R* ^2^	*P* value	*R* ^2^	*P* value
IL-13	0.249	<0.0001	0.289	<0.0001
IL-6	0.170	0.021	0.418	<0.0001
IL-5	0.139	0.061	0.257	0.0046
IL-4	0.456	<0.0001	0.329	<0.0001

Associations between variables were tested using the Spearman rank correlation test and expressed as Spearman correlation coefficients. *P* values of <0.05 were considered statistically significant.

### 3.3 Correlation of sB7-H3 and YKL-40 with severe MPP

**Figure 3:**
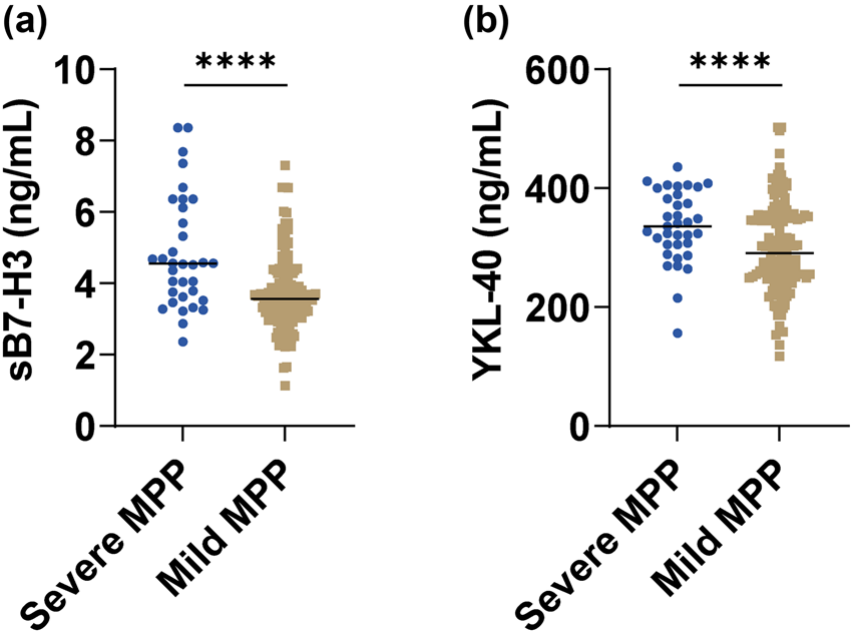
Correlation of sB7-H3 and YKL-40 with severe MPP. (a) sB7-H3, (b) YKL-40. ****P < 0.0001.

Of 182 children, 34 eventually progressed to severe MPP, of which 64.71% (22/34) occurred in the RMPP group. We compared sB7-H3 and YKL-40 levels in children with mild and severe MPP. As expected, there were higher sB7-H3 and YKL-40 levels in children with severe MPP (both *P* < 0.0001, Figure 3).

### 3.4 Correlation analysis of sB7-H3 and YKL-40 with RMPP

We included variables with significant differences in univariate analysis into binary logistic analysis. Statistical analysis identified CRP, D-dimer, IL-13, IL-6, IL-4, sB7-H3, and YKL-40, a total of seven variables. We excluded the following indicators: length of hospitalization, total fever duration, and NEP%. In addition, we adjusted for age and gender as confounders. Binary logistic regression analysis showed that sB7-H3 and YKL-40 were independent risk factors for RMPP (*P* < 0.05, Table 3). According to Hosmer and Lemeshow goodness of fit (*P* = 0.179), the model had a good fit.

**Table 3:** Binary logistic regression analysis for predicting factors associated with RMPP

Variables	*β*	S.E.	Wald	OR	95% CI	*P* value
sB7-H3	0.026	0.009	9.08	1.027	1.008–1.045	0.003
YKL-40	0.039	0.007	35.34	1.039	1.026–1.053	0

*β*, biased regression coefficient; S.E., standard error of the regression coefficient; OR, odd ratio; 95% CI, 95% confidence interval. *P* values of <0.05 were considered statistically significant.

### 3.5 ROC curves for predicting RMPP

**Figure 4:**
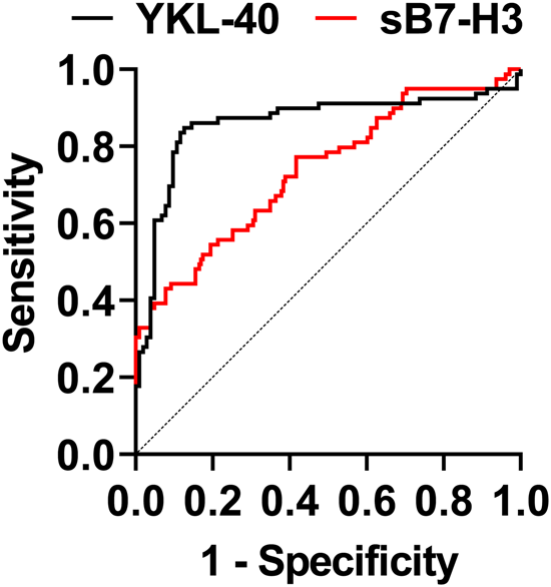
sB7-H3, YKL-40 ROC curves for predicting RMPP.

ROC curve analysis was performed to predict the critical values of sB7-H3 and YKL-40 for differentiating patients with RMPP from those with GMPP. sB7-H3 had an AUC value of 0.734, with a 95% CI of 0.664–0.811, *P* < 0.001 (Figure 4, Table 4). yKL-4 had an AUC value of 0.859, with a 95% CI of 0.794–0.923, *P* < 0.001 (Figure 4, Table 4). sB7-H3 and YKL-4 had critical values of 3.525 and 313.3 ng/mL.

**Table 4:** Critical values, specificity, and sensitivity of ROC curves for plasma sB7-H3 and YKL-40

	Cutoff	AUC (95% CI)	Sensitivity (%)	Specificity (%)	*P* value
sB7-H3 (ng/mL)	3.525	0.734 (0.664–0.811)	77.22	58.28	<0.001
YKL-40 (ng/mL)	313.3	0.859 (0.794–0.923)	88.61	87.38	<0.001

AUC, area under the curve; 95% CI, 95% confidence interval. *P* values less than 0.05 were considered statistically significant.

## 4 Discussion

MP has been associated with increasing death rates [33]. The incidence of RMPP continues to increase due to increased resistance to macrolide antibiotics [34]. There are several complications associated with RMPP, including necrotizing pneumonia, myocardial injury, endocarditis, organ and peripheral arterial embolisms, hepatic dysfunction, and autoimmune hemolysis [35]. Currently, it has been reported that the pathogenesis of the associated respiratory symptoms caused by MP is mainly lung cell injury and immune response injury [36,37]. Experimental and clinical evidence has also demonstrated that the severity of disease may be closely related to host immune response [38]. Therefore, early diagnosis of RMPP by exploring new biomarkers has a crucial role in reducing the incidence of RMPP and improving its prognosis.

This study encompassed 182 MPP patients, contrasting the clinical features of those with RMPP and GMPP. Under the diagnostic guidelines for RMPP, 79 individuals were identified with RMPP and 103 with GMPP. A statistical examination of the clinical traits of 182 children with MPP revealed that those in the RMPP group experienced longer hospital stays and prolonged fever compared to those in the GMPP group. Experimental results indicated elevated levels of NEP%, CRP, D-dimer, IL-13, IL-6, IL-5, IL-4, sB7-H3, and YKL-40 in the RMPP group compared to the GMPP group, potentially linked to the disease severity. Of the 182 children with MPP, 34 eventually progressed to severe MPP, 22 of which were from the GMPP group. Subsequent statistical evaluations revealed a strong correlation between RMPP and sB7-H3 levels of 3.525 ng/mL or more, as well as YKL-40 levels of 313.3 ng/mL or higher.

B7-H3 is a co-stimulatory molecule, which has been suggested to contribute to the immune dysfunction of the organism, the imbalance of homeostasis and the resulting immune dysfunction may cause inflammatory damage to the tissues of the organism. There is an overlap between RMPP and severe MPP in terms of clinical features, difficulty of treatment and poor prognosis. Increased plasma soluble B7-H3 levels can be used to assess the severity of patients with MPP [39]. Consistent with the results of that study, the present study also reconfirmed that severe MPP children have high plasma B7-H3 levels. However, the mechanism of B7-H3 in RMPP has not been reported. Resident lung dendritic cells likely contribute to the immunopathology of mycoplasma disease by generating mycoplasma-specific Th2 responses [40]. B7-H3 is known to promote the secretion of IL-4, IL-5, and IL-13 factors by providing to promote Th2 cell differentiation [41]. Previous studies have shown that B7-H3 promotes the levels of inflammatory factors such as IL-4, IL-5, and IL-13 in a bronchial asthma model through both TLR2-dependent and TLR2-independent signaling [42,43]. In the present study, we demonstrated that relative to GMPP, these inflammatory factors and sB7-H3 were elevated in patients with RMPP and a strong positive correlation with IL-4 was observed.

Many studies have shown that YKL-40 is involved in and regulates the pathophysiologic processes of several childhood respiratory diseases, such as bronchial asthma, allergic rhinitis, capillary bronchitis, pneumonia, and bronchopulmonary dysplasia [44,45]. YKL-40 levels are increased in serum and bronchoalveolar fluid of patients with viral pneumonia, and that the levels correlate with disease severity and prognosis [46]. However, no study has reported the role of blood YKL-40 in patients with RMPP. YKL-40 increases the number of dendritic cells and decreases T-cell apoptosis or increases T-cell survival in the lungs, which further activates and leads to the polarization of Th2-type cytokines and an increase in the number of Th2-type cells [47]. Furthermore, in systemic infectious patients, monocytes/macrophages, NEPs, and cancer cells all produce YKL-40, and IL-6 plays a key role in mediating the regulation of plasma YKL-40 levels during inflammation [48]. In the present study, we observed that YKL-40 levels were higher in children with RMPP compared to the GMPP group, and there was a significant positive correlation between YKL-40 and IL-6. In addition, YKL-40 was also significantly higher in children with severe MPP than in those with mild disease. Therefore, we suggest that YKL-40 promotes disease progression by mediating immune processes *in vivo*.

Although differences in CRP, LDH, and D-dimer levels between GMPP and RMPP have been reported in several studies, a combination of multiple metrics [49] is required to have discriminatory predictive value. We identified sB7-H3 and YKL-40 as independent correlates of RMPP. Further, both sB7-H3 and YKL-40 could effectively differentiate between GMPP and RMPP, and the value of YKL-40 in predicting RMPP was higher than that of sB7-H3. Therefore, YKL-40 has a significant value in the early prediction of RMPP, and the role of YKL-40 in the pathogenesis of RMPP needs to be further explored.

### 4.1 Limitations

This study has some limitations. First, since this is a retrospective study, there may be selection bias. Further prospective studies are recommended. Second, the number of patients included in this study was relatively small. Considering the complexity of Th1/Th2 cell imbalance in pneumonia, we considered the levels of Th2 cell-secreted factors in patients with MPP and could not determine the levels of serum factors secreted by Th1 cells. Considering the number of patients studied, in the binary logistic analysis, we excluded high-level variables and included only age and gender in the correction for confounders. Finally, the reliability of the results was better served by choosing more efficient and accurate assays and instruments for the measurement of factor levels.

## 5 Conclusion

sB7-H3 and YKL-40 levels were independent risk factors for RMPP. Children with RMPP had significantly elevated levels of sB7-H3 and YKL-40. YKL-40 had a higher predictive value for RMPP than sB7-H3; therefore, sB7-H3 testing facilitates the early diagnosis of RMPP.
